# Promoting parenting strategies to improve tooth brushing in children: design of a non-randomised cluster-controlled trial

**DOI:** 10.1186/s12903-019-0902-6

**Published:** 2019-09-06

**Authors:** Maddelon de Jong-Lenters, Monique L’Hoir, Erica Polak, Denise Duijster

**Affiliations:** 1 Department of Cariology Endodontology Pedodontology, Academic Center for Dentistry Amsterdam, University of Amsterdam and VU University, Gustav Mahlerlaan 3004, 1081LA Amsterdam, The Netherlands; 2 Uitblinkers referral pediatric dental practice, Diamantlaan 174a, 2332GR Leiden, The Netherlands; 3 Wageningen University, devision of Human Nutrition and Health Helix, Stippeneng 4, Building 124, 6708 WE Wageningen, The Netherlands; 4 GGD North & East Gelderland, Rijksstraatweg 65, 7231 AC Warnsveld, The Netherlands; 5 Opvoedpoli Amsterdam Noord, Rode Kruisstraat 32, 1025KN Amsterdam, The Netherlands; 6 Department of Social Dentistry, Academic Center for Dentistry Amsterdam, University of Amsterdam and VU University, Gustav Mahlerlaan 3004, 1081LA Amsterdam, The Netherlands

**Keywords:** Dental caries, Prevention, Intervention, Parenting, Tooth brushing, Self-efficacy

## Abstract

**Background:**

Tooth brushing with fluoride toothpaste is a key recommendation in evidence-based guidelines for caries prevention. Parents generally have sufficient knowledge to practice tooth brushing for their child, yet many experience barriers to actually implement the behaviour. Common barriers are associated with difficult child behaviour, stress, poor family organisation and management of routines. These underlying determinants of tooth brushing behaviour should be addressed in caries-preventive interventions.

The ‘Uitblinkers’ intervention is a semi-structured interview method developed for oral healthcare professionals (OHPs), with the aim to improve the practice of twice daily tooth brushing in children. The interview method focusses on ^1)^ identifying parents’ barriers to tooth brushing, and ^2)^ promoting parenting strategies (related to tooth brushing) to tackle the identified barriers. The intervention applies principles from learning theory, including stimulus control, operant conditioning and authoritative parenting. This paper describes a study protocol to evaluate the effect of the intervention.

**Methods:**

This non-randomised cluster-controlled trial will be conducted in 40 general dental practices in The Netherlands. Intervention practices will implement the intervention in addition to care as usual, while control practices will only provide care as usual. From each dental practice, a random sample of 3 to 4-year-old children will be recruited. The intervention consists of three sessions between an OHP and parent, in which parenting strategies for identified barriers are discussed. The primary study outcome is children’s dental caries experience after 24 months. Secondary outcomes include parents’ self-efficacy in brushing their children’s teeth, tooth brushing frequency in children and children’s dental plaque scores. Differences in outcomes between the intervention and control group will be assessed using logistic and negative binomial regression. The feasibility of the intervention will be assessed through process evaluation.

**Discussion:**

Findings of this study will ascertain whether promoting parenting strategies is a successful method to improve tooth brushing in children and to prevent childhood dental caries in a clinical dental setting.

**Trial registration:**

This trial is registered with the Netherlands National Trial Register (registration date: 7 September 2018; trial registration number: NTR7469).

**Electronic supplementary material:**

The online version of this article (10.1186/s12903-019-0902-6) contains supplementary material, which is available to authorized users.

## Background

The prevention of tooth decay in children seems fairly straightforward. The disease can largely be prevented by the use of fluorides, good oral hygiene and a non-cariogenic diet. There is overwhelming evidence that long-term exposure to low concentrations of fluoride is associated with a clear reduction in dental caries [1]. According to the World Health Organization (WHO), fluoride toothpaste is the most widespread and accepted form of fluoride use globally, and the most realistic and effective means to reduce the burden of tooth decay in populations [2]. Hence, twice daily tooth brushing with fluoride toothpaste by the parent as soon as the first primary teeth erupt is one of the key messages in evidence-based guidelines for caries prevention, including those of Public Health England [3] and the Dutch Ivory Cross [4].

The challenge however, lies in achieving personal compliance with this message. The conventional method to improve children’s oral hygiene, i.e. through dental health education and awareness raising, rarely leads to the intended behaviour change [5]. Systematic reviews confirmed the limited effectiveness of a purely educative approach in producing long-term oral hygiene improvements [6, 7], particularly when it is solely focussed on knowledge provision and tooth brushing instructions. Knowledge and technical skills are *necessary*, but seldom *sufficient* for sustained oral hygiene behaviour change.

Guidelines recommend that parents brush their children’s teeth and supervise tooth brushing until children are 10 years old [4]. Qualitative research with parents revealed that almost all parents recognize the importance of twice daily tooth brushing for their child [8, 9]. However, many parents reported barriers to actually adhere to the advice. Non-compliant child behaviour was often due to tantrums, pain during teething or tiredness of the child. Some parents avoided conflict in those situations than to persist on tooth brushing, which often meant engaging in very lively debates with their child. Other parents reported challenges with tooth brushing due to time constraints, a busy schedule or feelings of stress or fatigue. These findings highlight that simple behaviours such as tooth brushing are enmeshed in more complex daily habits, which are influenced by a range of child, parental and family-related factors.

Naturally, regular tooth brushing is more likely to be practiced in a supportive and organised home environment, where roles and boundaries are well-defined and routines are managed. Duijster et al. [10] showed that children from families that lack structure and routines are at higher risk of developing dental caries. In addition, Abegg et al. [11] demonstrated that a certain extent of flexibility is important to ensure regular tooth brushing. Furthermore, parenting practices influence how behavioural directions of the parent are delivered to and accepted by the child. Ineffective parenting, characterised by inconsistent and highly demanding discipline practices, evokes more resistance and non-compliance in children [12, 13]. This may also negatively affect children’s compliance towards twice daily tooth brushing. Effective parenting on the other hand, in terms of positive involvement (nurturance and sensitivity), positive reinforcement (encouragement and compliments) and problem-solving, has been associated with better oral hygiene and lower levels of dental caries in children [14–17].

In summary, many barriers to twice daily tooth brushing seem to revolve around the family environment. In this context, efforts to improve children’s oral hygiene should not narrowly focus on knowledge provision alone. Efforts should focus on the underlying determinants of twice daily tooth brushing and incorporate components of interventions that target parenting and broader family behaviour.

### The ‘Uitblinkers’ intervention

**Fig. 1:**
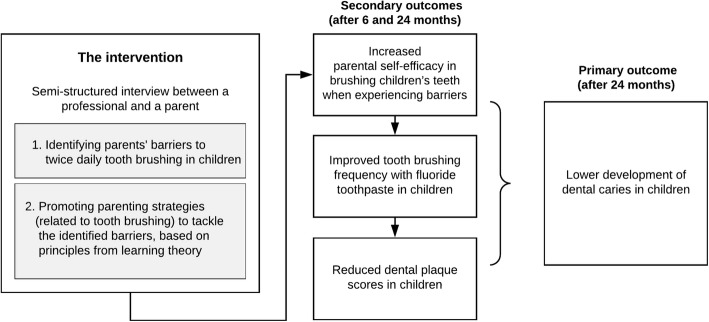
Components and hypothesised outcomes of the ‘Uitblinkers’ intervention

In 2017, the ‘Uitblinkers’ intervention (translation: brilliant stars) has been developed. The aim of this intervention is to improve the practice of twice daily tooth brushing in children to prevent dental caries on the long-term. The intervention is a semi-structured interview method primarily developed for a dental setting, targeting parents of 2 to 10-year-old children. The intervention focusses on identifying barriers that parents experience with brushing their children’s teeth, and promoting parenting strategies (related to tooth brushing) to tackle the identified barriers, using principles from learning theory. The hypothesis of the intervention is that the promotion of specific parenting strategies will increase parents’ self-efficacy (confidence) in brushing their children’s teeth when experiencing barriers, which in turn will lead to the improved practice of twice daily tooth brushing with fluoride toothpaste in children and reductions in children’s dental plaque scores. This is hypothesised to subsequently result in lower development of childhood dental caries. Figure 1 shows the components and hypothesised outcomes of the intervention.

#### Underlying theory – principles from learning theory

Principles from learning theory are applied to promote specific parenting strategies related to tooth brushing, with particular emphasis on stimulus control and operant conditioning. Stimulus control and operant conditioning are concepts from behavioural psychology, which argue that new behaviours or changes in behaviour are acquired through repetitive associations between stimuli and response [18].

Stimulus control refers to controlling stimuli before a behaviour occurs. Parenting skills related to stimulus control concentrate on enabling parents to create the conditions that stimulate, rather than hinder, desired behaviour in the child. This is done through structuring time and space, introducing ground rules and predictable routines, and through managing children’s problem behaviour by setting boundaries, giving clear instructions and the use of consistent measures [19].

Operant conditioning focusses on the relationship between a behaviour and its consequences. Operant conditioning can be defined as a learning process by which behaviours are modified by either (intermittent) reinforcement or a form of punishment [20, 21]. Parenting skills related to operant conditioning focus on teaching parents to positively reinforce desired behaviours of the child, e.g. through praise and reward. This will increase the likelihood that the child continues to show the desired behaviour. Undesired child behaviours should be decreased by ignoring the behaviour, which promotes extinction, or in some cases through punishment. Punishment could take several forms, including adding a negative stimulus (e.g. spanking), or removal of a positive stimulus (e.g. taking away a child’s toy). In child education, the use of the latter is being encouraged.

In addition to principles of stimulus control and operant conditioning, the intervention stimulates an authoritative parenting style, characterised by expression of warmth and sensitivity, and the use of clear communication about behavioural boundaries and expectations [22].

### Aim of the study

A study will be conducted to evaluate the ‘Uitblinkers’ intervention. The aim of the study is to assess the effect of the intervention on children’s dental caries experience after 24 months and on tooth brushing-related outcomes, including ^1)^ parents’ self-efficacy in brushing their children’s teeth, ^2)^ tooth brushing frequency in children and ^3)^ children’s dental plaque scores. A second objective is to conduct a process evaluation to assess the feasibility of the intervention, as well as oral healthcare professional’s (OPHs) and parents’ perceptions of the intervention. This paper describes the ‘Uitblinkers’ intervention and sets out the design of the study.

## Methods

### Design and study setting

The study is designed as a non-randomised cluster-controlled trial with a duration of 24 months. The study will be conducted in general dental practices located in The Netherlands. The medical ethical committee of the Vrije Universiteit in the Netherlands has provided ethical approval for this study (no. 17397).

### Study sample

#### General dental practices

The study will include 40 general dental practices: 20 practices in the intervention group and 20 in the control group. A convenience sampling method will be used to recruit dental practices for the intervention group. OHPs will be informed about the study at dental conferences and in-service training in The Netherlands, after which they are encouraged to voluntarily apply for participation. Practices will be eligible to participate if they have employed a certified dental therapist who is able to carry out and coordinate the study within their practice. Referral practices for paediatric dental care will be excluded from participation. All practices that apply will receive an information letter describing the procedures of the study, after which they will be asked to confirm their participation. Practices in both rural and urban regions will be included in the study.

For each participating dental practice in the intervention group, a matching control practice will be selected based on two factors: similar size of the patient population, with a maximum deviation in size of 500 registered patients; and same socioeconomic status (SES) of the region, as classified by the Dutch Central Bureau of Statistics on a scale from 1 (lowest SES) to 7 (highest SES) [23]. The size of the patient population gives an indication of the characteristics of a dental practice (e.g. solo or large group practice), and the SES of the region is indicative of the population’s (oral) health, with higher caries levels being reported in low SES regions [24]. Selected practices will be approached by e-mail, containing the information letter, and by telephone. As an incentive for participation, control practices will be offered training in the intervention method after the study is completed.

#### Children and their parent(s)

Each dental practice, both in the intervention and control group, will recruit a sample of at least ten 3 to 4-year-old children from their own patient population for inclusion in the study, using a simple random sampling method. Eligible children will be listed from the patient registry and given a random number by the coordinating dental therapist, using the website http://random.org. Subjects will be approached in numerical order until at least ten subjects are included. In cases of twins, only one child of the family will be selected. Children will be eligible to participate if they are healthy (ASA classification I) and between 36 and 59 months old at the time of inclusion. Exclusion criteria are:

- Children with a physical or mental disability,
- Children with a high caries activity (decayed, missing and filled teeth (dmft) ≥ 4), based on the patient dental record,
- Children with hypomineralised second primary molars,
- Children (or siblings of the child) who have been receiving treatment at a referral practice for paediatric dental care,
- Children of parents who do not speak and understand the Dutch language.

Three to 4-year-olds are selected for two reasons. First, the restriction of the age group reduces variation in dental caries levels, allowing better comparison of the intervention’s effect. Second, children are preferably targeted from a young age when oral health behaviours are still shaped and more amendable to change. However, in The Netherlands only 35% of two-year-olds are visiting the dentist, which increases to 95% when children reach the age of four [25]. Therefore, the choice was made to include 3 to 4-year-old children, increasing the number of young children that can be reached. Parents will be informed about the study aim and procedures, privacy and confidentiality regulations and their rights to withdraw at any time using an information letter (Additional file 1). Prior to participation, written informed consent will be obtained from the parents by the dental therapist.

#### Power calculation

The primary expected outcome of the study is a lower dental caries experience in the intervention group, compared to the control group, after 24 months. A power calculation indicated that a sample of 330 children will be required (165 children in each group) to detect a minimum difference of 2 decayed, missing and filled surfaces (dmfs) between the intervention and control group. This calculation is based on the following parameters: 80% power, a significance level of 5%, a mean dmfs of 6.9 ± 6.2 in 6-year-old children in the Dutch population [26], an expected loss to follow-up of 20 and 15% excess to account for clustering in the analysis. Further analyses indicated that a sample of 330 children will be amply sufficient to detect differences in tooth brushing-related outcomes.

### The intervention

All children, in both the intervention and control group, will receive care as usual, consisting of regular dental check-ups, dental health education following the Ivory Cross national guideline, and if indicated, preventive treatment (fluoride varnish and/or fissure sealants) and/or caries treatment (fillings, non-restorative caries treatment or extractions) [27]. Regular dental check-ups for young children are generally scheduled every 6 months, or at shorter intervals when children are identified as having an increased caries risk. Key oral health messages of the Ivory Cross national guideline include twice daily tooth brushing with fluoride toothpaste (500–750 ppm fluoride), reducing the frequency of carbohydrate intakes to three main meals and a maximum of four (healthy) snacks per day, and avoiding foods and drinks before or during bed time [4]. In addition to care as usual, parents of children in the intervention group will receive supplementary support to adhere to the advice of twice daily tooth brushing with fluoride toothpaste, following the ‘Uitblinkers’ intervention.

#### Development and procedure of the intervention

The ‘Uitblinkers’ intervention has been developed by a working group of researchers with expertise in paediatric dentistry, dental public health, educational psychology, behavioural therapy and pedagogy. It also uses (modified) components of the Dutch ‘BeeBOFT’ programme: an intervention to improve parenting skills for the prevention of childhood obesity [28]. The intervention is a semi-structured interview between an OHP (dentist, dental hygienist or dental therapist) and a parent. The interview focusses on two components: ^1)^ identifying possible barriers that parents may experience with brushing their children’s teeth, and ^2)^ exploration of possible parenting strategies to tackle the identified barriers using principles from learning theory. The intervention consists of a two-day training for OHPs and a toolkit (semi-structured script and cards) to guide the interview.

A core principle of the interview method is to create a positive, non-judgmental collaborative conversation between the OHP and the parent. This is created through expression of empathy, asking open-ended questions and through the provision of patient-centered advice, rather than the traditional authoritarian approach which heavily relies on a directive style of counselling (‘blaming the victim’). Evidence shows that people are more intrinsically motivated to change their behaviour if they feel autonomous, connected and actively involved in the counselling process [29]. Therefore, OHPs will be trained in empowering parents and stimulating their involvement in finding a suitable approach to tackle barriers related to tooth brushing. Many of the aforementioned conversation techniques are interview and counselling principles that are also applied in motivational interviewing (MI) [30] and in Egan’s ‘Skilled Helper’ model [31].

To identify barriers to tooth brushing, the OHP starts the interview by first asking about positive experiences (e.g. “What is going well with brushing your child’s teeth?”). This is to empower the parent and to avoid negative sentiment. This is followed by open-ended questions about the moments when tooth brushing is challenging. The OHP encourages the parent to explore their own barriers by asking for an elaboration, explicit examples or more details of challenging situations. Then, the OHP shows a set of nine cards, with on the front side barriers to twice daily tooth brushing (in text and in illustrations). The nine barriers are based on scientific literature [8, 9] and include “Tooth brushing is challenging …”

- “… when my child is heavily resisting or crying”
- “… when my child is too tired”
- “… when my child wants to brush by his/herself”
- “… because I don’t want to force my child against his/her will”
- “… when my child has pain”
- “… when it’s too busy (in the morning)”
- “… when it’s too busy (in the evening)”
- “… when I am tired”
- “… when I am stressed or pre-occupied”.

The cards and illustrations are designed to make parents aware of common barriers that are known to exist among parents, and to support the conversation especially for those who are low literate. The parent is subsequently asked to select the barrier he or she best identifies with.

After a barrier has been selected, the OHP and parent will discuss possible parenting strategies to address the specific barrier, whereby the OHP provides background information on the basics of stimulus control or operant conditioning in relation to tooth brushing. The strategies per barrier that are put forward by the OHP are described on the back-side of each card and summarised in Table 1. The OHP and parent aim to arrive at an agreed action plan, which will be documented for follow-up. Parents who are not able to name any barrier related to twice daily tooth brushing will be excluded from the study.

**Table 1:** Possible approaches to address parental barriers to twice daily tooth brushing, based on principles from learning theory

Barrier	Principle	Approach (summary)
“Tooth brushing is challenging when…”		
My child is heavily resisting or crying	Operant conditioning/authoritative parenting	• Ensure that tooth brushing in practiced on a regular basis, using small steps and affirmations to build towards a cooperative atmosphere. • Make parents aware that if tooth brushing is skipped because the child does not cooperate, the child is being rewarded for this unwanted behaviour. Hence, the unwanted behaviour is reinforced and will tend to continue. • Explain to parents that tooth brushing should happen at all times, even when the child is being uncooperative. Negative unwanted behaviours can be extinguished by ignoring them. Parents can gradually work towards a more cooperative atmosphere through a step by step approach (e.g. first brushing only two teeth), the use of compliments, and retaining a positive and calm attitude.
My child is too tired	Stimulus control	• Try to intervene before the unwanted behaviour occurs. • Ensure that tooth brushing is done before the child gets tired, by creating a predictable and efficient routine in the evening. • Make parents aware that children can become hyperactive and unmanageable when they are tired, which makes tooth brushing more challenging. • Help parents to think along how tooth brushing can be moved earlier in the evening, for example through a more efficient evening routine (e.g. already setting the table, or preparing foods), or by placing tooth brushing earlier in the sequence of evening activities (e.g. immediately after dinner).
My child wants to brush by his/herself	Operant conditioning/Chaining	• Make tooth brushing a collaborate activity; ensure that the parent retains control and that the child feels more autonomous. • Explain to parents how tooth brushing can be made into a collaborative activity: break the tooth brushing activity into small steps; allow the child to perform the simple steps (e.g. putting water in a cup, applying toothpaste on the brush); perform the actual brushing activity together while describing each step to the child; give compliments. The child is gradually allowed to perform more steps by itself. • Emphasise that the parent should always re-brush the child’s teeth.
I don’t want to force my child against his/her will	Operant conditioning/authoritative parenting/role modelling	• Ensure that tooth brushing in practiced on a regular, daily basis; the parent can function as a role model. • Make parents aware that if tooth brushing is skipped because the child does not want to brush, the child learns that tooth brushing can be avoided by saying no. Hence, this non-cooperative behaviour will be reinforced. • Explain to parents that tooth brushing should happen at all times, even when the child does not want to. If tooth brushing becomes a fact, children lose their motivation to argue. Parents can set a good example by letting the child copy the parent when brushing teeth and by giving compliments. Herewith, tooth brushing becomes a more fun and joint activity, which gradually reduces uncooperative behaviour.
My child has pain	Operant conditioning/authoritative parenting	• In case of actual pain (gingivitis; exfoliating teeth): Ensure that the painful/inflamed area is brushed twice a day with a soft tooth brush. Avoiding tooth brushing may worsen the pain. • In case of imaginary pain: Explain to parents that tooth brushing should happen at all times. Teach the child to deal with the pain, and let the child gradually experience that brushing is not as painful as they might have imagined, by building up tooth brushing in small steps (e.g. first brushing only two teeth or a not painful area in the mouth) and by giving compliments and praise.
It’s too busy (in the morning)	Stimulus control	• Help parents to structure time and space. • Ensure that tooth brushing is built into daily, activities, as a routine, creating a predictable and stable pattern in the morning. • Help parents find a moment in the morning where tooth brushing fits best in their sequence of activities and locations (e.g. in the bathroom before breakfast, in the kitchen after breakfast). • Advice parents that time can be saved by good preparations (e.g. already preparing the school lunch box in the prior evening).
It’s too busy (in the evening)	Stimulus control	• Help parents to structure time and space. • Ensure that tooth brushing is built into daily activities, as a routine way, creating a predictable and stable pattern in the evening. • Help parents find a moment in the evening where tooth brushing fits best in their sequence of activities and locations (e.g. immediately after dinner, before bedtime reading). • Make parents aware that tooth brushing becomes challenging once children are, for example, watching TV. Preferably tooth brushing is done before leisure time. Help parents to introduce ground rules and to apply them consistently.
I am tired	Stimulus control	• Ensure that tooth brushing is practiced on a regular basis as a routine. • Explain to parents that routines and regularity increases predictability of the behaviour, which reduced unwanted/uncooperative child behaviour and takes less energy from the parent.
I am stressed or pre-occupied	Authoritative parenting	• Ensure that stress from the parent is not transferred to the child, by staying calm and positive during the tooth brushing and bed time routine. • Explain to parents that children feel the stress of a parent (e.g. parents raising their voice, becoming a bit hard-handed). Children often respond to stress by copying the behaviour (e.g. shouting, showing resistance). This will make tooth brushing more challenging. • Advice parents to stay calm, not to raise their voice and to keep the atmosphere positive in order to facilitate tooth brushing for the child. The stress of the parent should not become the stress of the child.

#### Contact moments

The study consists of five contact moments for parents in the intervention group:

- T0.0: baseline - visit to the dental practice:
  Baseline data collection; first interview, including identification of a barrier, discussion of a possible strategy and agreement on an action point.
- T0.1: 1 month after T0.0 - visit to the dental practice (parent only):
  Second interview, including follow-up on the progress with the first action point, identification of a second barrier (if present), discussion of a possible strategy and agreement on a second action point.
- T0.2: 1 month after T0.2 – telephone recall:
  Telephone recall to follow-up on the progress with the first and second action points.
- T1: 6 months after T0.0 – visit to the dental practice:
  Follow-up data collection.
- T2: 24 months after T0.0 – visit to the dental practice:
  Follow-up data collection.

Where possible, the research visits at the dental practice will be combined with a regular dental check-up. Parents in the control group will visit the dental practice only at T0.0, T1 and T2 for baseline and follow-up data collection. The interval between T0.0 and T1 is allowed to deviate by 2 months, and the interval between T1 and T2 may vary with 3 months. If parents miss their appointment at T1 and T2, or at T2 only, they will be treated as dropouts. If they only miss the appointment at T1, missing data for T1 will be imputed. Missed telephone recalls will be documented to account for this in the analysis.

#### Training of oral healthcare professionals

The dental therapists participating in the intervention group will receive training in the intervention. The training will consist of three half-day training sessions led by the research team, and will include interactive lectures on learning theory, behavioural psychology, responsive parenting and interview and counselling techniques. Mock meetings will be conducted to practice the interview method using role-playing exercises. Dental therapists will also be encouraged to video-record themselves when practicing the method with a parent or colleague. These video recordings will function as learning material for the second and third training session in which the group will be asked to give constructive feedback to each other. All materials and theories discussed during the training will be made available for the dental therapists in the form of a syllabus.

Dental practices in both the intervention and control group will separately be informed about the study procedures, which includes information on the recruitment of participants and methods of data collection, as well as information on the provision of care as usual and dental health education according to national guidelines [4, 28]. It is expected that this is standard procedure for practices, but the importance of adherence to the guidelines for this study will be emphasised.

### Outcome measures

The primary outcome of this trial, children’s dental caries experience after 24 months, are measured at T2. Secondary outcomes, namely ^1)^ parents’ self-efficacy (confidence) in brushing their children’s teeth when experiencing barriers, ^2)^ tooth brushing frequency in children, and ^3)^ children’s dental plaque scores, are measured at T0.0, T1 and T2. The intervention is considered successful when a 25% reduction in dental caries experience is observed in the intervention group in comparison to the control group, which equals a reduction of 1.5 to 2.0 dmfs. Tooth brushing-related outcomes are assessed to provide additional information on the intervention’s effect and on the pathways by which the intervention might affect children’s caries experience. If one of more tooth brushing-related outcomes are significantly more favourable in the intervention group compared to the control group, but dental caries experience is not reduced by at least 25%, the intervention is not considered successful.

### Data collection

#### Tooth brushing-related outcomes

1. Parents’ self-efficacy in brushing their children’s teeth

A validated questionnaire from Finalyson et al. [32] will be used to measure parents’ self-efficacy in brushing their children’s teeth when experiencing barriers. The questionnaire consists of a nine-item measure which inquires parents about their confidence in making sure children’s teeth are brushed before bedtime when the parent is ^1)^ under a lot of stress, ^2)^ depressed, ^3)^ anxious, ^4)^ feeling like not having the time (too busy), ^5)^ tired, ^6)^ worrying about other things in life, ^7)^ bothered by their child crying, ^8)^ bothered because their child doesn’t stay still when wanting to brush, ^9)^ told by their child that he / she doesn’t feel like brushing right now (Additional file 2). Possible responses range from ‘1’ (not at all confident) to ‘4’ (very confident). Responses to the nine questions will be summed and a mean score is calculated to generate a total self-efficacy score.

2. Tooth brushing frequency in children

Parents will be requested to complete a self-administered questionnaire which includes questions on the frequency of brushing their child’s teeth (‘not every day’, ‘once a day’, ‘twice a day’ and ‘three times a day’), skipping tooth brushing when the child doesn’t want to brush (‘always’, ‘often’, ‘sometimes’, ‘never’) and the use of fluoride toothpaste (Additional file 2). The questions are selected from a validated questionnaire from Pine et al. [33] and have been forth and back-translated and tested in the Dutch context [10].

3. Children’s dental plaque scores

Dental plaque indices are used to assess an individual’s level of plaque control, providing an indication of the efficiency of tooth brushing and the state of oral hygiene [34]. In this study, dental plaque accumulation in children will be assessed on the vestibular surfaces of the central and lateral incisors in the upper and lower jaw. The dental hygienist or therapist will apply disclosing solution on the tooth surfaces using a cotton swab, after which the child is asked to rinse once. A digital white-light photograph will be taken of the teeth in an end-to-end position using a smartphone camera, using lip and cheek retractors to allow good view of the teeth. All images will be captured within 2 min of disclosure to minimise colouring variation. The images will be manually scored using the modified Quigley & Hein index [34]. Dental plaque will be scored on three sites of each tooth (distal, buccal, mesial) on a 6-point scale from no plaque (score ‘0’) to more than 2/3rd of surface covered with plaque (score ‘5’). A mean plaque score will be computed by calculating the sum of scores divided by the total number of scored tooth surfaces. All images will be scored by one trained and calibrated examiner from the research team, and 10% of the images will be scored by a second examiner to assess the inter-examiner reliability. Both examiners will be blind to whether the image is taken at T0.0, T1 or T2, and in an intervention or control practice.

#### Clinical outcome: children’s dental caries experience

At T2, a clinical oral examination will be performed to assess children’s dental caries experience. The clinical oral examination will only include a visual inspection and no x-rays will be taken. Dental caries status will be assessed using the merged International Caries Detection and Assessment System (ICDAS) coding system [35], which classifies dental caries into sound (‘0’), initial stage of decay (‘A’), moderate decay (‘B’) or extensive decay (‘C’). In addition, the dmfs-index will be scored according to WHO Basic Methods for Oral Health Surveys [36]. The oral examinations will be performed by one dentist from each control or intervention practice, after they have received training from a certified ICDAS trainer for calibration and standardisation purposes. The training will be organised 23 months after inclusion of the first participants. During the training, the dentists’ intra- and inter-examiner reliability will be assessed; a minimum weighted kappa of 0.60 for both the intra and inter-examiner reliability is required to certify as an examiner [37]. In case of lower reliability scores, more training will be provided. The dmft and dmfs at baseline will be extracted from personal dental health records from the dental practice, in order to compare differences in dental caries experience between intervention and control children at baseline, and to adjust for potential differences in the analysis.

#### Co-variates

Sociodemographic characteristics, including the child’s age, gender, parental education level, household income, family structure and ethnicity, will be collected using a questionnaire. The questionnaire also includes questions on other oral health behaviours, such as dietary factors, selected from the questionnaire from Pine et al. [32] (Additional file 2). These variables are collected because they are known moderators of the tooth brushing-related and clinical outcomes, and they therefore need to be adjusted for in the analyses. In addition, information will be extracted from dental records on the restorative and preventive treatments children received during the study, to allow to adjust for this in the analysis and interpretation of data.

#### Feasibility of the intervention (process evaluation)

Nine months after the training, a follow-up session will be organised for the dental therapists who are participating in the intervention group. During this session, semi-structured focus group interviews will be conducted to explore dental therapists’ perceptions about the feasibility of the intervention. The focus group interview will cover the following topics: experiences and challenges with the use of the interview method, perceived usefulness of the supporting materials (script and cards), their impressions regarding parents’ acceptance and appreciation of the approach, and suggestions for improvement of the intervention. Prior to the focus group interview, dental therapists are requested to complete a questionnaire on the same topics to supplement the qualitative data. The interview guide and questionnaire are presented in Additional files 3 and 4, respectively.

In addition, individual telephone interviews will be conducted with a random selection of 20 parents who received the intervention. Parents will be asked about their perceptions of the interview, and about whether or how it helped them to improve parenting strategies to facilitate tooth brushing for their children. The focus group and telephone interviews will be audio-recorded and transcribed verbatim.

#### Measures of fidelity

Fidelity is defined as the extent to which delivery of an intervention adheres to the protocol originally developed [38]. To measure fidelity, dental therapists in the intervention group will be requested to document the selected barriers and action points of participants. Questions on the implementation of the intervention are included in the questionnaire and focus group interview with dental therapists (see Additional files 2 and 3). In addition, the 20 parents who will be contacted for a telephone interview will be asked to give a description of their research visit, to determine whether the study protocol was followed as intended.

To assess whether the target group is reached, dental therapists in both intervention and control practices will be requested to document the number of subjects that agreed to participate out of the number of eligible subjects that were approached, as well as reasons not to participate. Non-participating parents will be asked two questions on their highest completed level of education and country of birth, to be able to assess potential selection bias.

#### Timeline

**Fig. 2:**
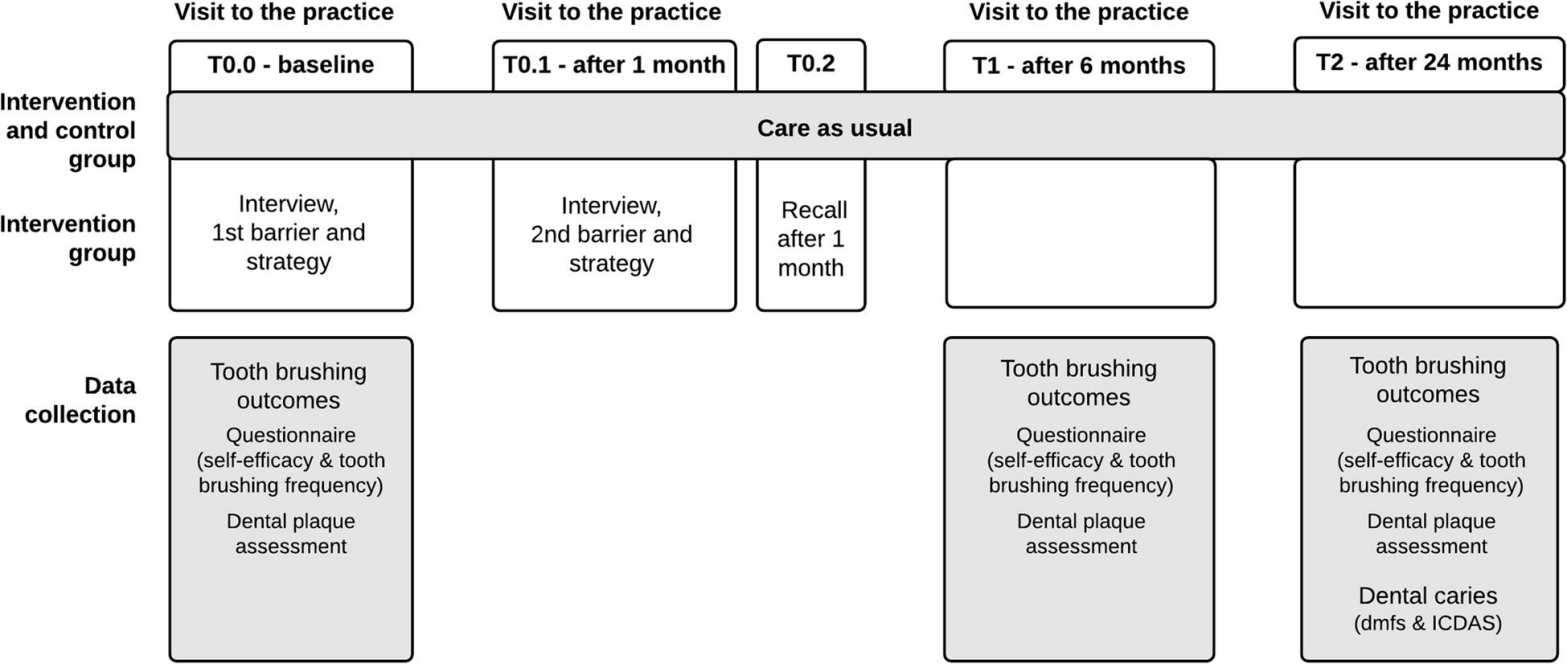
Timeline of the study

The timeline of the study, including the overview of research visits and data collection, is shown in Fig. 2.

### Statistical analysis

All data will be coded with a unique ID, and data will be securely stored at the Department of Social Dentistry at ACTA. The analysis will be performed in STATA. Differences in the primary outcome (dmfs and the combined ICDAS scores ‘B and C’ and ‘A, B and C’) between children in the intervention and control group at T2 will be analysed using zero-inflated Poisson or negative binomial regression. Baseline differences in children’s dmft scores (obtained from dental records) will be taken into account.

Differences in the three tooth brushing-related outcomes between children in the intervention and control group at T1 and T2 will be analysed using ordered logistic or logistic regression (for tooth brushing frequency in children; either treated as a ordinal variable or dichotomous variable, depending on the distribution of responses), and linear or negative binomial regression (for parental self-efficacy scores and children’s plaque scores).

All analysis will be adjusted for clustering of observations within dental practices. In case of significant differences in co-variates (moderators) between the intervention and control group at baseline (T0), the analysis will be adjusted accordingly for these co-variates, or in case of sufficient power, a sub-group analysis will be performed. An intention-to-treat analysis will be conducted to account for participants that will be lost to follow-up. Statistical significance is set at α = 5%.

Qualitative data from the focus group interviews and telephone interviews will be processed using the Maximised Qualitative Data Analysis programme (Verbi-software MAXQDA). Conventional content analysis will be used to identify emergent themes and concepts related to the feasibility and acceptability of the intervention.

### Blinding

The research team will be blind to whether the participants are assigned to the intervention or control group when scoring dental plaque and performing the data analysis. Study participants and OHPs providing the intervention and/or collecting data cannot be blinded.

## Discussion

This paper presents a study protocol to evaluate the process and effectiveness of the ‘Uitblinkers’ intervention in preventing dental caries in children. To the authors’ knowledge, this is the first intervention that is specifically directed towards improving parenting strategies related to tooth brushing using principles from learning theory. An asset of the intervention is that it was developed based on scientific analysis of important determinants of dental caries (‘change targets’), and the selection of theory- and evidence-based interventions from literature (‘behaviour change methods’), as recommended by the Intervention Mapping Approach of Bartholomew et al. [39]. A growing body of literature points to the important role of several family factors in children’s tooth brushing behaviour and subsequent oral health-related outcomes, such as maternal stress, parenting practices and broader family functioning [10–17, 40]. These family factors compose a subset of potentially modifiable determinants from the complex web of individual, familial, social and economic determinants of childhood dental caries that - when addressed - will likely translate to beneficial changes in children’s caries experience.

Thus far, no dental interventions had been developed that emphasise a broader family system framework to prevent childhood dental caries, by incorporating components that target parenting strategies, family organisation and routines as contexts of change. Yet, such a framework is promising, given the evidence that such interventions can be effective in the treatment and prevention of other childhood diseases, including childhood obesity. A systematic review on weight loss interventions [41] concluded that many programmes that incorporated components of parenting styles, parenting skills and child management principles showed a positive effect on weight loss in overweight children [42–45]. Furthermore, the Dutch ‘BeeBOFT’ intervention [28], which focusses on effective parenting strategies to prevent childhood obesity, has been associated with a higher likelihood of playing outside, having breakfast at the table and watching less TV [46]. The methods of ‘BeeBOFT’ may also prove successful in improving parenting strategies related to tooth brushing, and were therefore used as one of the bases to guide the selection and development of behaviour change methods of the study’s intervention.

A strength of this non-randomised cluster-controlled trial is the evaluation of the intervention’s impact on tooth brushing-related outcomes, as well as its clinical impact on children’s caries experience over a period of 24 months. Several process characteristics will also be assessed, such as the feasibility of the intervention as perceived by parents and OHPs, and information on implementation fidelity. This will provide insights that could be relevant for potential adaption and improvement of the intervention approach. Another strength is that the study will be conducted by OHPs in a real world clinical dental setting. The challenges and learnings that result from these real-setting experiences will be useful to facilitate future implementation. The participating dental practices will be located in both rural and urban regions, which benefits the generalisability of this study.

This study also has a few limitations that should be acknowledged. The method to recruit dental practices may cause selection bias. The study uses a convenience sample of dental practices in the intervention group, who are included based on voluntary application as opposed to randomisation. As a result, levels of engagement and motivation to provide preventive dental care may be higher in the intervention group. The generalisability of our study is further limited by excluding parents who do not understand the Dutch language and children with a very high caries activity (dmft ≥4 at the age of 3 years). Also, children who do not visit the dentist at the age of three and four will not be included in the study, which could potentially bias the sample towards more motivated parents and children. Furthermore, despite the use of validated questionnaires, parents may respond in a social desirable manner to questions related to self-efficacy and tooth brushing frequency. Lastly, there is a risk that dental therapists will drop out of the study. In this scenario, a colleague from the same dental practice will be requested and trained to collect follow-up data from the already included participants. The risk of contamination bias is expected to be low, given the clustered design of the trial, and the fact that control practices will not be informed about the practices that are in the intervention group and vice versa. Furthermore, control practices will not be able to provide the intervention without having received the supporting tools and training in methods and underlying theory.

Limitations of the intervention include that the method is designed for a dental setting, targeting children of 2 years or older, whilst tooth brushing behaviours are initiated at an earlier age. Furthermore, the intervention is specifically directed to improve tooth brushing behaviour and fluoride use, while dietary factors are equally important in the aetiology of dental caries. This decision was made because it is known from literature that dietary behaviour is highly complex and difficult to change in a clinical setting [47], while tooth brushing is a simpler and more delimited behaviour to change. If the intervention is proven feasible and effective, the intervention can be further developed in future research for use in high risk populations and/or for use in other care settings (e.g. baby well clinics), and through expanding its focus towards dietary behaviours.

In conclusion, this study will evaluate the effectiveness of the ‘Uitblinkers’ intervention in improving the practice of twice daily tooth brushing in children. Findings of this study will ascertain whether improving parenting strategies (related to tooth brushing) is a feasible and successful method to prevent childhood dental caries in a clinical dental setting.

## Additional files

Additional file 1: A. Information letter for parents (intervention group) – English. B. Information letter for parents (control group) – English. C. Information letter for parents (intervention group) – Dutch. D. Information letter for parents (control group) – Dutch. (PDF 163 kb)

Additional file 2: Questionnaire ‘Shine!’ study (English). (PDF 116 kb)

Additional file 3: Interview guide for dental therapists (English). (PDF 63 kb)

Additional file 4: Questionnaire for dental therapists (English). (PDF 110 kb)

## Data Availability

Results on the primary and secondary outcomes of this trial will be submitted for publication in national and international peer reviewed scientific journals. After data collection has been completed and results have been published, an anonymised dataset will be made publicly available at an appropriate data archive.

## Abbreviations

ASA: American Society of Anaesthesiologists
dmfs: Decayed, missing and filled surfaces
dmft: Decayed, missing and filled teeth
ICDAS: International Caries Detection and Assessment System
OHP: Oral healthcare professional
WHO: World Health Organization
