# Supplementary material for: Promoting parenting strategies to improve tooth brushing in children: design of a non-randomised cluster-controlled trial
Source: BMC Oral Health. 2019 Sep 6;19:210. doi: 10.1186/s12903-019-0902-6 (PMC6731582; doi:10.1186/s12903-019-0902-6)
Supplement: Supplementary file 1 — A. Information letter for parents (intervention group) – English. B. Information letter for parents (control group) – English. C. Information letter for parents (intervention group) – Dutch. D. Information letter for parents (control group) – Dutch. (PDF 163 kb) [file 12903_2019_902_MOESM1_ESM.pdf]

uitblinkers PROJECT

ACADEMISCH CENTRUM TANDHEELKUNDE AMSTERDAM
ACTA
GUSTAV MAHLERLAAN 3004
1081 LA AMSTERDAM

Appendix 1A. Information letter for parents (intervention group) – English

Dear parent, carer,

In the Netherlands, there are many young children with cavities in their teeth. Therefore, the University of Amsterdam is conducting research to improve the health of children's teeth. Your dental practice supports this research. We hope that you would also like to take part.

Tooth brushing twice a day is important for healthy teeth. Yet, this is easier said than done - sometimes tooth brushing in children can be challenging. Parents play an important role in brushing their child's teeth. Furthermore, the cooperation of your child itself is important. The purpose of this research is to study if extra guidance at the dentist can help parents to make tooth brushing easier and thus prevent cavities in your child.

What does participation mean for you?

The research consists of 4 visits to the dentist over a period of 2 years. If you participate in this study, your child will receive usual care at the dentist. In addition, you will receive extra guidance to help brush your child's teeth.

During the next dental check-up of your child, you will have a conversation with the dental therapist about barriers and tips to brush your child's teeth. He or she will use handy and supportive cards. This conversation takes approximately 15 minutes extra. We also ask you to fill in a short questionnaire. The dental therapist will also check whether your child's teeth are clean.

Four weeks later you will come back to the dentist's office. You will then have a follow-up conversation with the dental therapist. After one month, he or she will call you to ask about the progress and how things are going with tooth brushing.

At the check-up visit after 6 months and after 2 years, you will be asked to fill in a short questionnaire again. Your child's mouth will also be checked to see if it is clean and if there are any cavities.

All data collected will be used for the study.

What happens with the data?

The data collected about you and your child will be treated confidentially. The data will be processed in coded form.

Do I have to participate?

Your participation is completely voluntary. Furthermore, you have the right to withdraw at any time, without having to give a reason. The decision to withdraw will have no effect on the treatment of your child at the dentist.

You will soon be called to answer any questions you may have. You can also send an e-mail to m.lenters@acta.nl.

We hope you will take part!
Thank you in advance on behalf of the University of Amsterdam and your dental practice.

With kind regards,

Dr. Maddelon de Jong-Lenters
Pediatric dentist and researcher

Dr. Denise Duijster
Researcher

Appendix 1B. Information letter for parents (control group) – English

Dear parent, carer,

In the Netherlands, there are many young children with cavities in their teeth. Therefore, the University of Amsterdam is conducting research to improve the health of children's teeth. Your dental practice supports this research. We hope that you would also like to take part.

Tooth brushing twice a day is important for healthy teeth. Yet, this is easier said than done - sometimes tooth brushing in children can be challenging. Parents play an important role in brushing their child's teeth. Furthermore, the cooperation of your child itself is important. The purpose of this research is to study if extra guidance at the dentist can help parents to make tooth brushing easier and thus prevent cavities in your child.

What does participation mean for you?

You are selected to take part in the control group. If you participate in this study, your child will receive usual care at the dentist. The study consists of 3 visits to the dentist over a period of 2 years. These 3 visits coincide with the regular dental check-up. The visits include the next check-up and the check-up after 6 and 24 months.

During the 3 regular dental check-ups, you will be asked to fill in a short questionnaire. In addition, the dental therapist will check whether your child's teeth are clean. The dentist will also check your child's teeth for cavities. These data will be used for the study.

What happens with the data?

The data collected about you and your child will be treated confidentially. The data will be processed in coded form.

Do I have to participate?

Your participation is completely voluntary. Furthermore, you have the right to withdraw at any time, without having to give a reason. The decision to withdraw will have no effect on the treatment of your child at the dentist.

You will soon be called to answer any questions you may have. You can also send an e-mail to m.lenters@acta.nl.

We hope you will take part!

Thank you in advance on behalf of the University of Amsterdam and your dental practice.

With kind regards,

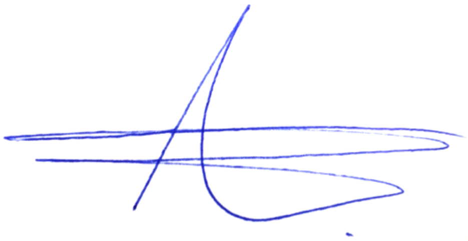

Dr. Maddelon de Jong-Lenters
Pediatric dentist and researcher

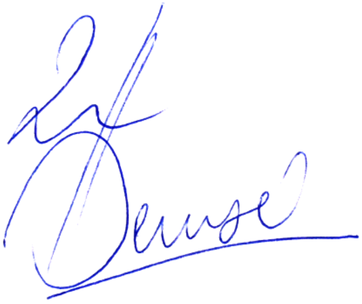

Dr. Denise Duijster
Researcher

Appendix 1C. Information letter for parents (intervention group) – Dutch

Geachte ouder, verzorger,

In Nederland zijn er veel jonge kinderen met gaatjes in de tanden en kiezen. Daarom wordt er op de Universiteit van Amsterdam onderzoek gedaan om de gezondheid van het gebit van kinderen te verbeteren. Uw tandartspraktijk doet hieraan mee. We hopen dat u hier ook aan wil meedoen.

Twee keer per dag tandenpoetsen is belangrijk voor een gezond gebit. Dit is gemakkelijker gezegd dan gedaan. Ouders spelen een grote rol bij het tandenpoetsen van hun kind. Verder is de medewerking van uw kind zelf van belang. Het doel van dit onderzoek is om te kijken of extra begeleiding bij de tandarts kan helpen om het poetsen makkelijker te maken en zo gaatjes bij uw kind te voorkomen.

Wat betekent meedoen voor u?

Het onderzoek bestaat uit 4 bezoeken bij de tandarts over een periode van 2 jaar. Als u meedoet aan dit onderzoek krijgt uw kind gewoon de zorg die u gewend bent. Daarnaast krijgt u extra begeleiding bij het tandenpoetsen van uw kind.

Tijdens de eerstvolgende controle van uw kind krijgt u een gesprek met de assistent over tandenpoetsen. Zij gebruikt hierbij handige en duidelijke kaartjes. Dit gesprek duurt ongeveer 15 minuten extra. Wij vragen u ook een korte vragenlijst in te vullen. Ook bekijkt de assistent of de tanden van uw kind schoon zijn.

Vier weken later komt u nog een keer naar de tandartspraktijk. U krijgt dan een vervolggesprek met de assistent van 15 minuten. Na een week belt de assistent u om te vragen hoe het gaat met het tandenpoetsen bij uw kind.

Bij de controles na 6 maanden en 2 jaar vult u weer een korte vragenlijst in. Ook wordt bij uw kind gekeken of de mond schoon is en of er eventueel gaatjes zijn.

Alle verzamelde gegevens worden voor het onderzoek gebruikt.

Wat gebeurt er met de gegevens?

De verzamelde gegevens over u en uw kind zullen vertrouwelijk worden behandeld. De gegevens worden gecodeerd verwerkt.

Moet ik meedoen?

Uw deelname is geheel vrijwillig. Verder mag u altijd zonder reden stoppen, ook tijdens het onderzoek. De beslissing om te stoppen zal geen enkel gevolg hebben voor de behandeling van uw kind bij de tandarts.

Binnenkort wordt u gebeld om eventuele vragen van uw kant te beantwoorden. U kunt ook zelf mailen naar m.lenters@acta.nl.

We hopen dat u meedoet!

Bij voorbaat dank namens de Universiteit van Amsterdam en uw tandartspraktijk.

Met vriendelijke groet,

Dr. Maddelon de Jong-Lenters
Kindertandarts en onderzoeker

Dr. Denise Duijster
Onderzoeker

ACADEMISCH
CENTRUM
TANDHEELKUNDE
AMSTERDAM
GUSTAV MAHLERLAAN 3004
1081 LA AMSTERDAM

Appendix 1D. Information letter for parents (control group) – Dutch

Geachte ouder, verzorger,

In Nederland zijn er veel jonge kinderen met gaatjes in de tanden en kiezen. Daarom wordt er op de Universiteit van Amsterdam onderzoek gedaan om de gezondheid vanhet gebit van kinderen te verbeteren. Uw tandartspraktijk doet hieraan mee. We hopen dat u hier ook aan wil meedoen.

Twee keer per dag tandenpoetsen is belangrijk voor een gezond gebit. Dit is gemakkelijker gezegd dan gedaan. Ouders spelen een grote rol bij het tandenpoetsen van hun kind. Verder is de medewerking van uw kind zelf van belang. Het doel van dit onderzoek is om te kijken of extra begeleiding bij de tandarts kan helpen om het poetsen makkelijker te maken en zo gaatjes bij uw kind te voorkomen.

Wat betekent meedoen voor u?

U bent ingedeeld in de controlegroep. Als u meedoet aan dit onderzoek krijgt uw kind gewoon de zorg die u gewend bent. Het onderzoek bestaat uit 3 bezoeken bij de tandarts over een periode van 2 jaar. Deze 3 bezoeken vallen samen met de controle. De bezoeken bestaan uit de eerstvolgende controle en de controle na 6 en 24 maanden.

Tijdens de 3 reguliere controlebezoeken vult u een korte vragenlijst in. Daarnaast bekijkt de assistent of de tanden van uw kind schoon zijn. Ook controleert de tandarts het gebit van uw kind op gaatjes. Deze gegevens worden voor het onderzoek gebruikt.

Wat gebeurt er met de gegevens?

De verzamelde gegevens over u en uw kind zullen vertrouwelijk worden behandeld. De gegevens worden gecodeerd verwerkt.

Moet ik meedoen?

Uw deelname is geheel vrijwillig. Verder mag u altijd zonder reden stoppen, ook tijdens het onderzoek. De beslissing om te stoppen zal geen enkel gevolg hebben voor de behandeling van uw kind bij de tandarts.

Binnenkort wordt u gebeld om eventuele vragen van uw kant te beantwoorden. U kunt ook zelf mailen naar m.lenters@acta.nl.

We hopen dat u meedoet!

Bij voorbaat dank namens de Universiteit van Amsterdam en uw tandartspraktijk.

Met vriendelijke groet,

Dr. Maddelon de Jong-Lenters
Kindertandarts en onderzoeker

Dr. Denise Duijster
Onderzoeker
