# Supplementary material for: Promoting parenting strategies to improve tooth brushing in children: design of a non-randomised cluster-controlled trial
Source: BMC Oral Health. 2019 Sep 6;19:210. doi: 10.1186/s12903-019-0902-6 (PMC6731582; doi:10.1186/s12903-019-0902-6)
Supplement: Supplementary file 2 — Questionnaire ‘Shine!’ study (English). (PDF 116 kb) [file 12903_2019_902_MOESM2_ESM.pdf]

Appendix 2 - Questionnaire 'Shine!' study (English)

1. ID Code ___________________________________

2. Child's date of birth ____day / _____ month / _____year

3. Child's gender: ☐ male ☐ female

4. What is your relation to the child?
- ☐ biological mother
- ☐ adoptive mother
- ☐ stepmother
- ☐ foster mother
- ☐ grandmother
- ☐ biological father
- ☐ adoptive father
- ☐ stepfather
- ☐ foster father
- ☐ grandfather
- ☐ other, namely ___________________________

TOOTH BRUSHING

1. How often are your child's teeth usually brushed?
- ☐ not every day
- ☐ once a day
- ☐ twice a day
- ☐ three times a day

2. How old was your child when he/she first started having his/her teeth brushed?
- ☐ Under 1 year
- ☐ 1 year – under 2 years
- ☐ 2 year – under 3 years
- ☐ 3 years or over
- ☐ Cannot remember

3. Who brushes you child's teeth?
- ☐ only a parent / caregiver (please go to question 6)
- ☐ only the child
- ☐ both parent / caregiver and the child

4. Do you check whether your child has brushed his/her teeth?
- ☐ always
- ☐ often
- ☐ sometimes
- ☐ never

5. Do you re-brush your child's teeth?

- ☐ always
- ☐ often
- ☐ sometimes
- ☐ never

6. Is your child allowed to skip tooth brushing, for example when he/she is tired or doesn't feel like brushing?

- ☐ always
- ☐ often
- ☐ sometimes
- ☐ never

7. Does your child use toothpaste?

- ☐ yes, always
- ☐ yes, sometimes
- ☐ no (please go to question 8)

What brand of toothpaste does your child usually use?

Name of brand ________________________________

8. "How confident are you about making sure your child's teeth are brushed before bedtime when you are…?

	very confident	fairly confident	not so confident	not at all confident
a. under a lot of stress	☐	☐	☐	☐
b. depressed	☐	☐	☐	☐
c. anxious	☐	☐	☐	☐
d. feeling like you don't have the time (too busy)	☐	☐	☐	☐
e. tired	☐	☐	☐	☐
f. worriying about other things in your life	☐	☐	☐	☐
g. bothered by your child crying	☐	☐	☐	☐
h. bothered because your child doesn't stay still when you want him or her to brush	☐	☐	☐	☐
i. told by your child that he or she does not feel like brushing right now	☐	☐	☐	☐

<u>FOODS AND DRINKS</u>

1. How many days a week does your child have breakfast?

- ☐ 1 day a week
- ☐ 2 days a week
- ☐ 3 days a week
- ☐ 4 days a week
- ☐ 5 days a week
- ☐ 6 days a week
- ☐ 7 days a week

2. Many children take a drink to bed with them either to have before they go to sleep, or during the night. How often does your child have something to drink in bed or during the night?

☐ always
☐ often
☐ sometimes
☐ never (please go to question 4)

3. When your child has a drink in bed or during the night, what does he/she usually have?
(Please tick as many boxes as necessary)

☐ fruit juice
☐ lemonade
☐ milk
☐ milk with sugar or honey
☐ milk drinks (e.g. chocolate milk)
☐ water
☐ tea
☐ fizzy drinks
☐ other, namely _______________________

4. Thinking about food, how often does your child eat in bed or during the night?

☐ always
☐ often
☐ sometimes
☐ never (please go to question 6)

5. When your child has something to eat when going to sleep or during the night, what does he/she usually have?*(Please tick as many boxes as necessary)*

☐ sweet biscuits
☐ savoury / plain biscuits or crackers
☐ cakes
☐ crisps or savoury snacks
☐ cakes
☐ fruit
☐ sweets or chocolate
☐ sandwiches
☐ other, namely_________________________

6. How often does your child <u>eat</u> something between meals (for example, fruit or biscuits)?

☐ never
☐ less than once a day
☐ 1-2 times a day
☐ 3-5 times a day
☐ 6-10 times a day
☐ more than 10 times a day

7. How often does your child <u>drink</u> something between meals (for example, fruit juice, milk; water and tea without sugar not included)?

☐ never
☐ less than once a day
☐ 1-2 times a day
☐ 3-5 times a day
☐ 6-10 times a day

☐ more than 10 times a day

BACKGROUND INFORMATION

1. **What's the child's country of birth**___________________

2. **What's the country of birth of the mother of the child?** ____________________________

3. **What's the country of birth of the father of the child?** _____________________________

4. **What language is usually spoken at home?** ______________________________

5. What is the highest completed level of education of the mother?

☐ no education
☐ elementary school
☐ secondary school (VBO, VMBO, MAVO)
☐ secondary school (HAVO, VWO)
☐ lower vocational education (LBO/MBO)
☐ higher vocational education (HBO)
☐ university
☐ I don't know (I am not the mother of the child)

6. What is the postal code of the child's home address (first four numbers only)?
____

7. How many children do you have?

☐ 1 child
☐ 2 children
☐ 3 children
☐ 4 or more children

8.Is this your first child, second child, etc?

☐ first
☐ second
☐ third
☐ fourth or more

Please take the time to make sure you have answered all questions.

Thank you very much for your help!

Appendix 2 – Vragenlijst Uitblinkers-onderzoek (Nederlands)

1. Naam kind ___________________________________

2. Geboortedatum kind ____dag / _____ maand / _____ jaar

3. Geslacht kind: ☐ jongen ☐ meisje

4. Wat is uw relatie tot het kind?
☐ biologische moeder
☐ adoptief moeder
☐ stiefmoeder
☐ pleegmoeder
☐ grootmoeder
☐ anders, namelijk ___________________________
☐ biologische vader
☐ adoptief vader
☐ biologische vader
☐ pleegvader
☐ grootvader

<u>TANDEN POETSEN</u>

1. Hoe vaak worden de tanden van uw kind gepoetst?
☐ niet iedere dag
☐ altijd 1 keer per dag
☐ meestal 1 keer, soms 2 keer per dag
☐ meestal 2 keer, soms 1 keer per dag
☐ altijd 2 keer per dag
☐ meestal 2 keer, soms 3 keer per dag
☐ meestal 3 keer, soms 2 keer per dag
☐ altijd 3 of meer keer per dag

2. Hoe oud was uw kind toen zijn/haar tanden voor het eerst werden gepoetst?
☐ minder dan 1 jaar
☐ 1 tot 2 jaar
☐ 2 tot 3 jaar
☐ Kan ik mij niet herinneren

3. Wie poetst de tanden van uw kind?
☐ alleen de ouder / verzorger (ga door naar vraag 6)
☐ alleen het kind
☐ ouder / verzorger en kind

4. Controleert u of uw kind zijn/haar tanden heeft gepoetst?
☐ altijd
☐ meestal
☐ af en toe
☐ nooit

5. Poetst u de tanden van uw kind na?

☐ altijd
☐ meestal
☐ af en toe
☐ nooit

6. Mag uw kind het tandenpoetsen wel eens overslaan (bijvoorbeeld als hij/zij moe is of geen zin heeft)?

☐ altijd
☐ meestal
☐ af en toe
☐ nooit

7. Gebruikt uw kind tandpasta bij het poetsen?

☐ ja, altijd
☐ ja, soms
☐ nee

Zo ja, welk merk tandpasta koopt u meestal voor het gebruik van uw kind?

Merknaam ....

8. "Hoe zeker bent u ervan dat het u lukt om voor het slapen gaan de tanden van uw kind te poetsen, als u…?

	Heel zeker	Redelijk zeker	Niet zo zeker	Helemaal niet zeker
a. Stress heeft	☐	☐	☐	☐
b. Zich depressief voelt	☐	☐	☐	☐
c. Gespannen voelt	☐	☐	☐	☐
d. Geen tijd heeft (te druk)	☐	☐	☐	☐
e. Moe bent	☐	☐	☐	☐
f. Zich druk maakt om andere dingen in het leven	☐	☐	☐	☐
g. Last heeft van uw huilend kind	☐	☐	☐	☐
h. Last heeft dat uw kind niet meewerkt met het poetsen	☐	☐	☐	☐
i. Hoort van uw kind dat hij/zij geen zin heeft om te poetsen	☐	☐	☐	☐

VOEDING

1. Hoeveel dagen per week ontbijt uw kind?

☐ 1 dag per week
☐ 2 dagen week
☐ 3 dagen per week
☐ 4 dagen per week
☐ 5 dagen per week
☐ 6 dagen per week
☐ 7 dagen per week

2. Veel kinderen nemen wat te drinken mee naar bed, zodat zij iets kunnen drinken tijdens de nacht. Hoe vaak neemt uw kind iets te drinken in bed?

- ☐ nooit
- ☐ soms
- ☐ meestal
- ☐ altijd

3. Wat drinkt uw kind meestal wanneer hij/zij iets te drinken heeft in bed? (kruis alle hokjes aan die van toepassing zijn)

- ☐ vruchtensap
- ☐ vruchtenlimonade
- ☐ melk
- ☐ melk met suiker of honing
- ☐ melk bevattende dranken (bijv. chocolademelk)
- ☐ water
- ☐ thee
- ☐ frisdrank
- ☐ anders, namelijk ……………………………………

4. Hoe vaak eet uw kind iets in bed gedurende de nacht?

- ☐ iedere dag
- ☐ de meeste dagen
- ☐ af en toe
- ☐ nooit

5. Wat eet uw kind meestal als hij/zij iets te eten heeft in bed? (kruis alle hokjes aan die van toepassing zijn)

- ☐ koekjes
- ☐ fruit
- ☐ boterhammen (zoet)
- ☐ cakes
- ☐ snoep of chocolade
- ☐ chips of hartige snacks
- ☐ anders, namelijk …………………………

6. Hoe vaak per dag <u>eet</u> uw kind iets tussen de maaltijden door (bijvoorbeeld snoep, een koek, fruit, etc)?

- ☐ nooit
- ☐ minder dan 1 keer
- ☐ 1-2 keer
- ☐ 3-5 keer
- ☐ 6-10 keer
- ☐ meer dan 10 keer

7. Hoe vaak per dag <u>drinkt</u> uw kind iets tussen de maaltijden door (bijv. fruitsap, wicky, frisdrank, siroop, thee met suiker. Water en thee zonder suiker niet meegerekend)?

- ☐ nooit
- ☐ minder dan 1 keer
- ☐ 1-2 keer
- ☐ 3-5 keer
- ☐ 6-10 keer
- ☐ meer dan 10 keer

ACHTERGRONDINFORMATIE

1. **In welk land is het kind geboren?**____

2. **Wat is het geboorteland van de moeder van het kind?** ___________________________

3. **Wat is het geboorteland van de vader van het kind?** _____________________________

4. **Welke taal wordt er thuis gesproken?** _____________________________

5. Wat is de hoogste opleiding die de moeder van het kind heeft afgemaakt?

- ☐ geen onderwijs gehad
- ☐ basisschool
- ☐ middelbare school (VBO, VMBO, MAVO)
- ☐ middelbare school (HAVO, VWO)
- ☐ Lager/middelbaar beroepsonderwijs (LBO/MBO)
- ☐ Hoger beroepsonderwijs (HBO)
- ☐ Universiteit
- ☐ weet ik niet (Ik ben niet zelf de moeder van het kind)

6. Wat is de postcode van uw thuisadres (de eerste 4 cijfers)? _______________________

7. Hoeveel kinderen heeft u?

- ☐ 1 kind
- ☐ 2 kinderen
- ☐ 3 kinderen
- ☐ 4 of meer kinderen

8. Uw hoeveelste kind is dit?

- ☐ eerste
- ☐ tweede
- ☐ derde
- ☐ vierde of meer

Neemt u alstublieft nog even kort de tijd om er zeker van te zijn dat u alle vragen heeft ingevuld.

Hartelijk bedankt voor uw hulp!
