# Supplementary material for: Promoting parenting strategies to improve tooth brushing in children: design of a non-randomised cluster-controlled trial
Source: BMC Oral Health. 2019 Sep 6;19:210. doi: 10.1186/s12903-019-0902-6 (PMC6731582; doi:10.1186/s12903-019-0902-6)
Supplement: Supplementary file 3 — Interview guide for dental therapists (English). (PDF 63 kb) [file 12903_2019_902_MOESM3_ESM.pdf]

Appendix 3 – Interview guide for dental therapists (English)

<u>Feasibility</u>

Time and planning

- What are your experiences with implementing the Shine! Method in your practice? What are barriers and what are facilitators?
- How much time does it take to apply the Shine! method?

Conversation techniques

- How did the training in the Shine! method affect your counselling style and the way you're having conversations with parents?
- How did you experience having conversations with parents about their personal home situation and barriers to tooth brushing?

Script and the cards with barriers

- How did you experience working with the script and cards? Did the cards facilitate or hinder the conversation, and how?
- Did parents recognize themselves in one or more barriers, and were any other barriers mentioned?
- What recommendations do you have to improve the script and / or cards?

Parenting strategies and coming to an agreed action point

- What are your experiences with discussing possible parenting strategies to tackle selected barriers? What went well, and what could have been better?

<u>Training</u>

- What are the most important things you learnt and remembered from the training?
- In your opinion, did you receive enough training to be able to apply the Shine! method in practice? If not, what else is needed, and how can the training be improved?

<u>Experiences of parents and potential efficacy</u>

- What were the reactions of parents who received the Shine! intervention? What did they like and what did they not like?
- What were parents' reactions to receiving a telephone recall after the interview?
- What is your impression of the effectiveness of the Shine! intervention if you compare it with traditional dental health education and instruction? What elements do you think work well, and what don't?

<u>Recruitment of participants and data collection</u>

- What are your experiences with recruiting patients for the study?
- What were the main reasons for parents not to participate?
- How did you experience collecting data for the study?

<u>General</u>

- After the study has been completed, do you think you would continue using the Shine! method in your dental practice? Why or why not, and which elements would you continue to use or not?
- In general, what recommendations do you have for improvement of the Shine! intervention?

Appendix 3 – Interview guide voor preventie-assistenten (Nederlands)

Uitvoerbaarheid

Tijd & planning

- Was de gespreksvoering goed uit te voeren in de praktijk? Waarom wel, of niet?
- Hoeveel tijd kost het om dit gesprek te voeren?
- Hoe zijn de afspraken ingepland in jullie agenda?
- Hoeveel patiënten hebben jullie kunnen doen?
- Hoeveel driejarigen hebben er in die periode de tandarts bezocht?

Manier van gespreksvoering

- Zijn jullie op een andere manier het gesprek aangegaan met ouders? Zo ja, wat hebben jullie anders gedaan, en hoe was dit voor jullie?
- Hoe was het om meer open in gesprek te gaan met ouders, en door te vragen naar de thuissituatie?

Het script en de kaarten met barrières

- Hoe was het voor jullie om met de kaarten te werken?
- Hielpen de kaarten bij het gesprek, of werd het er lastiger door. Waarom?
- Herkenden ouders zich in de kaarten en konden ze makkelijk kiezen?
- Werden er nog andere barrières genoemd?
- Hoe was het om volgens het semigestructureerde script te werken?
- Hebben jullie suggesties hoe we het script en de kaarten kunnen verbeteren?

Opvoedkundige principes en komen tot een aanpak

- De aanpak per barrière is gebaseerd op opvoedkundige principes, waaronder stimulus controle en operant conditioneren. Hoe was het voor jullie om een aanpak te bespreken op basis van de opvoedkundige principes. Wat ging er goed? Wat was er lastig?

Training

- Wat zijn de belangrijkste punten die jullie uit de training hebben gehaald?
- Was de training voldoende om met de gesprekstechniek aan de slag te gaan, of is meer oefening en theorie nodig?
- Zo ja, hoe zou de training kunnen worden verbeterd?

Ervaringen van ouders en effectiviteit

- Wat waren de reacties van ouders met wie jullie dit gesprek hebben gevoerd?
- Hoe ervaarden ouders de extra tijd die het gesprek kostte?
- Wat vinden ouders ervan dat ze achteraf worden gebeld?
- Hebben jullie indruk dat deze nieuwe gespreksmethodiek beter werkt om ouders te helpen met poetsen? Zo ja / Zo niet, waarom?

Werving

- Hoe was het om ouders voor het onderzoek te werven?
- Wat liep goed, en waar liepen jullie tegenaan?
- Wat waren redenen voor ouders om wel of niet mee te doen?
- Hoe ging het om de data te verzamelen?
- Wat zijn jullie ervaringen met het nemen van de plakfoto's.

Tot slot:

- Denken jullie de gesprekstechniek na dit onderzoek te blijven uitvoeren in de praktijk? Waarom / welke elementen wel/ niet?
- Welke aanbevelingen hebben jullie voor verandering / verbetering?
