# Supplementary material for: Promoting parenting strategies to improve tooth brushing in children: design of a non-randomised cluster-controlled trial
Source: BMC Oral Health. 2019 Sep 6;19:210. doi: 10.1186/s12903-019-0902-6 (PMC6731582; doi:10.1186/s12903-019-0902-6)
Supplement: Supplementary file 4 — Questionnaire for dental therapists (English). (PDF 110 kb) [file 12903_2019_902_MOESM4_ESM.pdf]

# Appendix 4 – Questionnaire for dental therapists (English)

## GENERAL

1. Name ___________________________
2. What is your age? ________________ years
3. Do you work in a…? (please tick multiple answers if necessary)
   ☐ Group practice
   ☐ Solo practice
4. How many years have you been working as a dental therapist?__ ______________ years
5. What kind of educational training have you received to become a dental therapist
   ☐ Vocational training for dental therapists (MBO)
   ☐ I am trained in the dental practice

## THE SHINE! STUDY

6. How many patients have you included for the Shine! study? __________________ patients
7. On average, how much time did it take you to have a conversation with a parent, following the Shine! method? _____________minutes

Below are a number of statements about the 'Shine!' method. Please indicate to what extent you agree with the statements. We are interested in your opinion and experiences, so there are no good or wrong answers.

| **Feasibility** | Fully agree | Agree | Neutral | Disagree | Fully disagree | N.A. |
|---|---|---|---|---|---|---|
| 8. I am using a different style of counselling since I have received training in the Shine! method. | ☐ | ☐ | ☐ | ☐ | ☐ | ☐ |
| 9. The Shine! method takes too much time. | ☐ | ☐ | ☐ | ☐ | ☐ | ☐ |
| 10. I am asking more open-ended questions since I received training in the Shine! method. | ☐ | ☐ | ☐ | ☐ | ☐ | ☐ |
| 11. Having a conversation following the Shine! method feels unnatural to me. | ☐ | ☐ | ☐ | ☐ | ☐ | ☐ |
| 12. I already applied many conversation techniques of the Shine! method before the training. | ☐ | ☐ | ☐ | ☐ | ☐ | ☐ |
| 13. Conversations with parents are more positive when using the Shine! method. | ☐ | ☐ | ☐ | ☐ | ☐ | ☐ |
| 14. I find it difficult to ask parents about their personal home situation during the interview. | ☐ | ☐ | ☐ | ☐ | ☐ | ☐ |
| 15. The Shine! method is innovative. | ☐ | ☐ | ☐ | ☐ | ☐ | ☐ |
| **Cards with barriers and the script** | Fully agree | Agree | Neutral | Disagree | Fully disagree | N.A. |

| | | | | | | |
|---|---|---|---|---|---|---|
| 16. The cards with barriers are a useful tool to support the conversation with parents. | ☐ | ☐ | ☐ | ☐ | ☐ | ☐ |
| 17. Parents could easily recognize themselves in one or more barriers. | ☐ | ☐ | ☐ | ☐ | ☐ | ☐ |
| 18. I find it difficult to use the cards during the conversation. | ☐ | ☐ | ☐ | ☐ | ☐ | ☐ |
| 19. The cards made it easier for parents to identify a barrier to tooth brushing. | ☐ | ☐ | ☐ | ☐ | ☐ | ☐ |
| 20. Some parents thought the cards were a bit strange. | ☐ | ☐ | ☐ | ☐ | ☐ | ☐ |
| 21. The script is a useful tool to guide the conversation. | ☐ | ☐ | ☐ | ☐ | ☐ | ☐ |

| **Training and parenting strategies** | Fully agree | Agree | Neutral | Disagree | Fully disagree | N.A. |
|---|---|---|---|---|---|---|
| 22. More training is needed on the intervention's underlying theory of parenting (stimulus control and operant conditioning). | ☐ | ☐ | ☐ | ☐ | ☐ | ☐ |
| 23. It is clear to me what possible parenting strategies I can discuss with a parent, depending on the chosen barrier. | ☐ | ☐ | ☐ | ☐ | ☐ | ☐ |
| 24. More training days are needed in order to be able to apply the Shine! method in practice. | ☐ | ☐ | ☐ | ☐ | ☐ | ☐ |
| 25. I would like to have more knowledge on what parenting strategies I can discuss for a certain barrier. | ☐ | ☐ | ☐ | ☐ | ☐ | ☐ |
| 26. I feel that I have been able to apply the Shine! method as it was explained to me during the training. | ☐ | ☐ | ☐ | ☐ | ☐ | ☐ |

| **Experiences of parents** | Fully agree | Agree | Neutral | Disagree | Fully disagree | N.A. |
|---|---|---|---|---|---|---|
| 27. Parents thought the Shine! method took too much time. | ☐ | ☐ | ☐ | ☐ | ☐ | ☐ |
| 28. Parents reacted positively to having a conversation about barriers to tooth brushing. | ☐ | ☐ | ☐ | ☐ | ☐ | ☐ |
| 29. At the end of the Shine! interview, parents went home with a concrete action plan to tackle their identified barrier to tooth brushing. | ☐ | ☐ | ☐ | ☐ | ☐ | ☐ |
| 30. Parents felt uncomfortable about me asking questions about their personal situation and barriers to tooth brushing. | ☐ | ☐ | ☐ | ☐ | ☐ | ☐ |

| **Own experiences and potential efficacy** | Fully agree | Agree | Neutral | Disagree | Fully disagree | N.A. |
|---|---|---|---|---|---|---|
| 31. I expect that the Shine! method is more succesful in changing tooth brushing behaviour than conventional dental health education. | ☐ | ☐ | ☐ | ☐ | ☐ | ☐ |
| 32. Asking parents about barriers to tooth brushing allows me to give more tailored advice. | ☐ | ☐ | ☐ | ☐ | ☐ | ☐ |

| | | | | | | | |
|---|---|---|---|---|---|---|---|
| 33. | The Shine! method is too complex to implement in dental practice. | ☐ | ☐ | ☐ | ☐ | ☐ | ☐ |
| 34. | I would like to continue using the Shine! method after the study has been completed. | ☐ | ☐ | ☐ | ☐ | ☐ | ☐ |

35. Apart from the 9 barriers on the cards, did parents experience any other barriers to tooth brushing? If so, what barriers were mentioned?
_______________________________________________________________________________
_______________________________________________________________________________
_______________________________________________________________________________
___

36. Do you have recommendations on how the Shine! method can be improved?
_______________________________________________________________________________
_______________________________________________________________________________
_______________________________________________________________________________
_______________________________________________________________________________

**Thank you very much for your help!**

Appendix 4 – Vragenlijst voor preventie-assistenten (Nederlands)

## ALGEMEEN

1. Naam ___________________________

2. Leeftijd ________________ jaar

3. In wat voor praktijk werkt u? (meerdere antwoorden mogelijk)
   - ☐ Groepspraktijk
   - ☐ Solopraktijk

4. Hoe lang bent u al werkzaam als preventie-assistent?________________ jaar

5. Wat voor opleiding tot preventie-assistent heeft u gehad?
   - ☐ MBO opleiding voor preventie-assistent
   - ☐ Opgeleid in een tandartspraktijk

## HET UITBLINKERS ONDERZOEK

6. Bij hoeveel patiënten heeft u de Uitblinkers-methode toegepast? ________ patiënten

7. Hoeveel tijd nam een gesprek volgens de Uitblinkers-methode gemiddeld in beslag? ________ minuten

Hieronder staan enkele stellingen over de Uitblinkers-methode. Wilt u aangeven in hoeverre u het met deze stellingen eens bent? Het gaat om uw mening en ervaringen; er zijn dus geen goede of foute antwoorden!

| **Uitvoerbaarheid** | Volledig eens | Mee eens | Neutraal | Oneens | Volledig oneens | N.v.t. |
|---|---|---|---|---|---|---|
| 8. Door de Uitblinkers-training ben ik op een andere manier gaan voorlichten | ☐ | ☐ | ☐ | ☐ | ☐ | ☐ |
| 9. Het toepassen van Uitblinkers-methode kost te veel tijd | ☐ | ☐ | ☐ | ☐ | ☐ | ☐ |
| 10. Ik stel meer open vragen aan ouders sinds de Uitblinkers-training | ☐ | ☐ | ☐ | ☐ | ☐ | ☐ |
| 11. Het voeren van een gesprek volgens de Uitblinkers-methode voelt (nog) onnatuurlijk | ☐ | ☐ | ☐ | ☐ | ☐ | ☐ |
| 12. Veel gesprekstechnieken van de Uitblinkers-methode paste ik hiervoor al toe | ☐ | ☐ | ☐ | ☐ | ☐ | ☐ |
| 13. Gesprekken met ouders verlopen positiever met de Uitblinkers-methode | ☐ | ☐ | ☐ | ☐ | ☐ | ☐ |
| 14. Ik vind het moeilijk om bij ouders door te vragen naar de persoonlijke thuissituatie | ☐ | ☐ | ☐ | ☐ | ☐ | ☐ |
| 15. De Uitblinkers-methode is vernieuwend | ☐ | ☐ | ☐ | ☐ | ☐ | ☐ |
| **Kaarten met barrières en script** | Volledig | Mee | Neutraa | Oneens | Volledi | N.v.t. |

| | eens | eens | l | | g oneens | |
|---|---|---|---|---|---|---|
| 16. De kaarten met hobbels bieden een goede ondersteuning voor het gesprek | ☐ | ☐ | ☐ | ☐ | ☐ | ☐ |
| 17. Ouders konden zich makkelijk herkennen in de kaarten met barrières | ☐ | ☐ | ☐ | ☐ | ☐ | ☐ |
| 18. Ik vond het lastig om de kaarten te gebruiken in het gesprek | ☐ | ☐ | ☐ | ☐ | ☐ | ☐ |
| 19. De kaarten maakten het makkelijker voor ouders om een poets-barrière te noemen. | ☐ | ☐ | ☐ | ☐ | ☐ | ☐ |
| 20. Sommige ouders vonden de kaarten maar raar | ☐ | ☐ | ☐ | ☐ | ☐ | ☐ |
| 21. Het script is een prettig hulpmiddel (leidraad) om het gesprek met ouders te voeren | ☐ | ☐ | ☐ | ☐ | ☐ | ☐ |

| **Training en opvoedkundige adviezen** | Volledig eens | Mee eens | Neutraal | Oneens | Volledig oneens | N.v.t. |
|---|---|---|---|---|---|---|
| 22. In de training moet meer aandacht worden besteed aan de opvoedkundige theorie (stimulus controle en operant conditioneren) | ☐ | ☐ | ☐ | ☐ | ☐ | ☐ |
| 23. Het is voor mij duidelijk welke opvoedkundige adviezen ik het beste kan bespreken met de ouder, afhankelijk van de barrière | ☐ | ☐ | ☐ | ☐ | ☐ | ☐ |
| 24. Er zijn meer dan 2 trainingsdagen nodig om met de Uitblinkers-methode aan de slag te kunnen gaan | ☐ | ☐ | ☐ | ☐ | ☐ | ☐ |
| 25. Ik heb behoefte aan meer kennis over welke opvoedkundige adviezen ik moet geven bij een gekozen barrière | ☐ | ☐ | ☐ | ☐ | ☐ | ☐ |
| 26. Het is me gelukt de Uitblinkers-methode toe te passen zoals in de training is uitgelegd | ☐ | ☐ | ☐ | ☐ | ☐ | ☐ |

| **Ervaringen van ouders** | Volledig eens | Mee eens | Neutraal | Oneens | Volledig oneens | N.v.t. |
|---|---|---|---|---|---|---|
| 27. Ouders vonden de Uitblinkers-methode te veel tijd kosten | ☐ | ☐ | ☐ | ☐ | ☐ | ☐ |
| 28. Ouders reageerden positief op het feit dat er een gesprek met ze werd gevoerd over barrières bij poetsen | ☐ | ☐ | ☐ | ☐ | ☐ | ☐ |
| 29. Na het Uitblinkers-gesprek hadden ouders een concreet actieplan om hun poets-barrière aan te pakken | ☐ | ☐ | ☐ | ☐ | ☐ | ☐ |
| 30. Ouders vonden het onprettig dat ik vragen stelde over hun thuissituatie en barrières bij poetsen | ☐ | ☐ | ☐ | ☐ | ☐ | ☐ |

| **Eigen ervaring en effectiviteit** | Volledig eens | Mee eens | Neutraal | Oneens | Volledig oneens | N.v.t. |
|---|---|---|---|---|---|---|
| 31. Ik verwacht dat de Uitblinkers-methode beter werkt dan standaard voorlichting om poetsgedrag te verbeteren | ☐ | ☐ | ☐ | ☐ | ☐ | ☐ |

| | | | | | | | |
|---|---|---|---|---|---|---|---|
| 32. | Door te vragen naar barrières bij poetsen, kan ik beter afgestemde adviezen aan ouders geven | ☐ | ☐ | ☐ | ☐ | ☐ | ☐ |
| 33. | De Uitblinkers-methode is te complex voor de praktijk | ☐ | ☐ | ☐ | ☐ | ☐ | ☐ |
| 34. | Ik wil de Uitblinkers-methode na het onderzoek blijven toepassen in de praktijk | ☐ | ☐ | ☐ | ☐ | ☐ | ☐ |

35. Zijn er nog andere barrières bij poetsen door ouders genoemd?

______________________________________________________________________________
______________________________________________________________________________
___________________________________________________________________________

36. Heeft u aanbevelingen hoe we de Uitblinkers-methode kunnen verbeteren?

______________________________________________________________________________
______________________________________________________________________________
______________________________________________________________________________
______________________________________________________________________________

**Veel dank voor uw inzet en voor het invullen van deze vragenlijst!**
